# Phenotypes and clinical significance of circulating CD4^+^CD25^+^ regulatory T cells (Tregs) in patients with acute-on-chronic liver failure (ACLF)

**DOI:** 10.1186/1479-5876-10-193

**Published:** 2012-09-15

**Authors:** Jiezuan Yang, Ping Yi, Li Wei, Zherong Xu, Yunbo Chen, Lingling Tang, Lanjuan Li

**Affiliations:** 1 State Key Laboratory for Diagnosis and Treatment of Infectious Diseases, the First Affiliated Hospital, College of Medicine, Zhejiang University, Hangzhou 310003, China; 2 Department of Geriatric, the First Affiliated Hospital, College of Medicine, Zhejiang University, Hangzhou, 310003, China

## Abstract

**Background:**

CD4^+^CD25^+^ regulatory T cells (Tregs) play an important role in maintaining immunological tolerance to self and foreign antigens. T cell receptors (TCR) reflect the composition and function of T cells. It is not universally agreed that there is a relationship between CD4^+^CD25^+^ Treg frequency and the severity of acute-on-chronic liver failure (ACLF). The repertoire of TCR beta chain variable (TCRBV) regions of peripheral Tregs in ACLF patients is not well understood.

**Methods:**

Human PBMCs were separated and sorted into CD4^+^CD25^+^ Treg subsets using density gradient centrifugation and magnetic activated cell sorting (MACS). The CD4^+^CD25^high^ Treg frequency in peripheral blood of ACLF and chronic hepatitis B (CHB) patients was measured by flow cytometry. The molecular profiles of TCRBV CDR3 were determined using gene melting spectral pattern (GMSP) analysis. TCRBV gene families were cloned and sequenced when the GMSP profiles showed a single-peak.

**Results:**

CD4^+^CD25^high^ Treg prevalence in peripheral blood of ACLF patients is increased significantly compared to healthy donors (HDs) (*P* < 0.01) and CHB patients (*P* < 0.01). The prevalence of CD4^+^CD25^high^ Tregs in ACLF or CHB patients is positively correlated with HBV DNA load. The TCRBV11, BV13.1, BV18, BV20 are the most prevalent TCRBV in CD4^+^CD25^+^ Tregs in ACLF and CHB patients. In addition, the CDR3 motifs were relatively conserved in these four TCRBV gene families.

**Conclusions:**

The CD4^+^CD25^high^ Tregs prevalence in peripheral blood is indicative of disease severity in ACLF or CHB patients. The relatively conserved TCRBV20 CDR3 motif “TGTGHSPLH” and TCRBV11 CDR3 motif “VYNEQ” may be used in helping diagnosis and treat patients with ACLF.

## Background

Hepatitis B virus (HBV) is responsible for chronic infection. Despite a reduction in new HBV infections since the introduction of vaccination in the early 1990s, there are approximately 350 million chronic HBV carriers worldwide, and HBV remains an important cause of liver disease in developed countries [1]. HBV infection can lead to a spectrum of liver diseases, including chronic asymptomatic HBV carrier (AsC), chronic hepatitis B (CHB), liver cirrhosis (LC) and hepatocellular carcinoma (HCC) or acute-on-chronic liver failure (ACLF) [2,3]. HBV-related ACLF is an acute decompensation of chronic liver disease due to precipitating events such as upper gastrointestinal (UGI) bleeding, ischemia, or additional superimposed liver injury due to alcohol, hepatotoxic drugs, surgical procedures, or reactivation of viral hepatitis [4]. ACLF has a relatively high mortality and liver transplantation serves as the most promising treatment for ACLF [5-7]. However, a serious lack of livers limits the utility of liver transplantation. Although the role of T-cell immunity in ACLF pathogenesis is receiving more attention, it is not well understood.

Recent studies have shed light on CD4^+^CD25^+^ regulatory T cells (Tregs) that actively maintain immune tolerance to both self and non-self-antigens by suppressing an aggressive T-cell response [8]. Studies indicate that an increase of circulating CD4^+^ CD25^+^ Tregs are associated with persistent HBV infection, due to down-regulated HBV-specific T cells [9,10]. The occurrence of ACLF often represents a complicated state of host immune dysregulation, in which exacerbated innate immune responses and aberrant adaptive immune responses may play an important role in mediating hepatic inflammation and liver necrosis. However, it is still controversial as to how the peripheral frequency of CD4^+^CD25^+^ Treg changes in patients with HBV-related ACLF [11,12].

The T cell receptor (TCR) repertoire can reflect the function, status, and composition of T cell populations *in vivo*. Characterization of the TCR repertoire of CD4^+^CD25^+^ Treg subsets from hepatitis B patients may clarify its role in ACLF pathogenesis. Furthermore, understanding TCR expression during disease progression, including for infectious diseases, may aid diagnosis and treatment [13]. In this study, we examined the frequency of peripheral CD4^+^CD25^+^ Tregs from HBV-infected ACLF patients, and the TCRBV CDR3 repertoire in Tregs from ALCF and CHB patients. A relationship between Treg frequency and HBV DNA load in ACLF or CHB patients was observed.

## Materials and methods

### Subjects

Patients with HBV-related ACLF (n = 25) and patients with CHB (n = 30) were admitted to the Department of Infectious Disease, the First Affiliated Hospital, College of Medicine, Zhejiang University, between February and June 2011. Healthy donors (n = 30) were selected as controls and matched for sex ratio and age with the patient groups. Written informed consent was obtained from all subjects prior to enrollment. The study was conducted according to the guidelines of the Declaration of Helsinki. The Zhejiang University medical ethics committee approved all procedures involving human subjects.

The diagnostic standards for patients with ACLF is set by the Consensus Recommendation on ACLF issued by the Asian Pacific Association for the Study of the Liver (APASL) [14], and the Diagnostic and Treatment Guidelines for Liver Failure [15]. ACLF patients were categorized as follows: (a) a history of chronic hepatitis or well-compensated cirrhosis with the presence of serum HBsAg ≥ 6 months; (b) recent development of severe jaundice with total bilirubin ≥ 171 μmol/L plus increasing international normalized ratio (INR) ≥ 1.5 or decreasing prothrombin activity (PTA) ≤ 40%; and (c) recent development of coagulopathy, obvious ascites, hepatic encephalopathy, hepatorenal syndrome [16]. Patients with CHB fulfilled the definitions described in detail elsewhere [11]. Patient characteristics at the time of the study are shown in Table 1. Patients co-infected with human immunodeficiency virus (HIV), hepatitis A virus (HAV), hepatitis C virus (HCV), hepatitis D virus (HDV), or hepatitis E virus (HEV), were excluded. Patients treated with anti-viral or immunomodulatory drugs within the previous 6 months, or suffering from a complication of spontaneous bacterial peritonitis (SBP) were excluded too. Peripheral venous blood samples were obtained after liver disease was diagnosed. The anti-viral treatment was applied together with conventional medical treatment, after the blood samples were obtained. Among the ACLF patients, 14 cases treated with entecavir (Bristol-Myers Squibb) 0.5 mg/d, and the remaining 11 cases with lamivudine (GlaxoSmithKline) 100 mg/d.

**Table 1:** Characteristics of HBV patients and healthy controls

	**ACLF n = 25**	**CHB n = 30**	**HCs n = 30**
Age (years)	43 ± 10	38 ± 7	36 ± 7
Gender (M/F)	16/9	21/9	18/12
ALT (IU/L)	327 ± 215	223 ± 124	18 ± 9
TBiL (μmol/L)	410.0 ± 150.2	133.2 ± 51.2	12.3 ± 3.7
HBV DNA(lgcps/mL)	5.3 ± 1.2	5.7 ± 1.6	ND
HBeAg (positive/negative)	12/13	18/12	ND
HBV genotype	15B, 10C	18B, 12C	ND

These values expressed as mean ± SD, unless otherwise indicated.

Normal values: *ALT*, ≤ 40 IU/L; *TBiL*, ≤ 21 μmol/L.

*ACLF*, acute-on-chronic liver failure; *ALT*, alanine transaminase; *CHB*, chronic hepatitis B; *DNA*, deoxyribonucleic acid; *HBV*, hepatitis B virus; *HCs*, healthy controls; *TBiL*, Total bilirubin; *ND*, no detected.

### Biochemical and serological markers and quantification of HBV DNA

Biochemical tests of liver function examined serum alanine amino transferase (ALT), aspartate amino transferase (AST), total bilirubin (TBiL), and creatinine (CREA) using an automated biochemical analyzer (AEROSET, Abbott, Chicago, IL, USA). Prothrombin activity (PTA) and international normalized ratio (INR) were derived from prothrombin time (PT) and determined using an automated coagulation analyzer (Sysmex CA-500 Series, Siemens, Deerfield, IL, USA). Qualitative assessment of HBV markers (HBsAg/anti-HBs, HBeAg/anti-HBe, anti-HBc) was determined using commercial enzyme immunoassay kits (LJ biology, Shanghai, China). Serum HBV DNA load was quantified and HBV genotypes were determined using commercial kits as described previously [17,18], with a minimal detection standard of 500 copies/mL.

### Peripheral blood mononuclear cell (PBMCs) isolation and flow cytometric analysis

PBMCs were isolated from 10 ml fresh EDTAK_2_ anti-coagulant-treated blood using Ficoll-Paque (StemCell Technologies Inc., Vancouver, Canada) density gradient separation. To determine the frequency of the CD4^+^CD25^+^ Treg population, 100 μL whole blood was incubated with fluorochrome-conjugated antibodies (BD Bioscience, Franklin Lakes, NJ, USA) to T cell surface markers CD4 (FITC-conjugated anti-CD4 antibody) and CD25 (PE-conjugated anti-CD25 antibody) at 4°Cfor 30 min. Red blood cells were lysed, stained, and fixed in 1% paraformaldehyde. Stained cells were washed twice with sheath fluid, and the percentage of CD4^+^CD25^+^ Tregs in the total CD4^+^ lymphocyte population was analyzed using flow cytometry (Beckman Coulter Fc500 MPL, Coulter, Fullerton, USA), and isotype-matched control antibodies.

### Preparation of CD4^+^CD25^+^ regulatory T cells and flow cytometric analysis

CD4^+^CD25^+^ Tregs were isolated from fresh PBMCs. Briefly, CD4^+^ T cell subsets were negatively selected using a CD4^+^ T cell biotin-antibody cocktail and anti-biotin microbeads. CD4^+^CD25^+^ T cells were further isolated from CD4^+^ T cells using anti-CD25 antibody-coated magnetic beads (Miltenyi Biotec, Bergisch Gladbach, Germany) according to the manufacturer’s instructions. The purity of CD4^+^CD25^+^ Tregs was ≥ 90% (data not shown), as determined by CD4/CD25 flow cytometric analysis using surface staining. For intracellular staining, the purified of CD4^+^CD25^+^ Tregs were treated with Fix & Perm Reagent (Coulter) after labelling with surface antibodies, and then incubated with anti-Foxp3 (eBioscience, San Diego, CA, USA), and the following steps as described above. The data were analyzed using MXP software (Coulter). The CD4^+^CD25^+^ Tregs were followed total RNA extracted, when the percentages of intracellular FoxP3-positive T cells in CD4^+^ T-cell subsets from ACLF or CHB patients were about 80%. In addition, the average frequency of CD127^low^ expressed on these CD4^+^CD25^+^ Tregs is approximately 80% (data no shown).

### RNA extraction and cDNA synthesis

Total RNA was extracted from CD4^+^CD25^+^ Tregs using TRIzol® (Invitrogen, Carlsbad, California, USA) or RNeasy® Mini Kit (Qiagen, Hilden, Germany) according to the manufacturer’s instructions. RNA was quantified using a NanoDrop® ND-2000 spectrophotometer (Thermo Fisher Scientific, Wilmington, Delaware, USA), and integrity was checked electrophoretically (28S and 18S). Total RNA was immediately reverse transcribed to cDNA using the RevertAid First Strand cDNA Synthesis Kit (MBI, Fermentas, EU) per manufacturer’s instructions. Briefly, ~1-5 μg total RNA was reverse transcribed with OligodT_18_ in a 20 μL reaction volume and stored at −20°C.

### GMSP assay of TCRBV in CD4^+^CD25^+^ tregs to identify skewed TCRBV repertoire

The GoTaq® qPCR Master Mix (with Rox™ dye) was used as PCR kit (Promega, Madison, WI, USA). A master mix of 20 μl for each of the 28 reactions containing 0.5 μM reverse primer TCRBC, and 50 ~ 150 ng template cDNA was prepared. For family specific TCRBV amplification, the corresponding forward primer (TCRBV1 ~ 24) was added to a final concentration of 0.5 μM. The real time PCR reaction parameters were as follows: 2 min at 95°Cto activate the GoTaq enzyme, followed by 45 cycles at 95°Cfor 15 s, 56.5°Cfor 20 s, and 72°Cfor 25 s with a final extension at 72°Cfor 8 min. The melting curve for 24 TCRBV gene families was determined by plotting the first negative derivative of the decrease in fluorescence signal as a function of temperature (−dF/dT) versus temperature (Tm), generating gene melting spectral patterns (GMSPs) as previously reported [19].

A skewed clonal expansion was determined using the GMSP profile of each TCRBV family using Opticon Monitor 3.0 and the MJ Opticon 2 DNA engine (Bio-Rad, Hercules, CA, USA). GMSP profiles were in one of two categories: 1) “Oligoclonal expansion”, or 2) “Monoclonal”, as defined previously [20].

### Cloning and sequencing

If a TCRBV gene family demonstrated a monoclonal GMSP profile (single peak), the sample was selected for cloning and sequencing to determine the degree of homogeneity within the CDR3 region. Briefly, PCR products were reamplified using GoTaq DNA polymerase (Promega, Madison, WI, USA) and touchdown PCR. The parameters were: pre-incubation at 95°Cfor 2 min, 95°Cfor 30 s, 60°Cfor 40 s, 72°Cfor 45 s, for 8 cycles with annealing temperature decreasing 0.5°Cper cycle, and 95°Cfor 45 s, 56°Cfor 45 s, 72°Cfor 50 s, for 27 cycles. The cycling was followed by a terminal elongation step at 72°Cfor 8 min.

Purified PCR products were ligated into pGEMT-T using the pGEM-3Z Cloning Kit (Promega, USA) according to the manufacturer’s instructions. Cloning details have been described previously [21]. The plasmid DNA was sequenced using an ABI3730 DNA Sequencer (Applied Biosystems, Foster City, USA). The resulting sequences were compared against a standard TCRBV gene database ( http://www.imgt.org).

### Statistical analysis

All data were analyzed using SPSS software version 16.0 (SPSS Inc., Chicago, USA). TCRBV families were analyzed using the Kruskal–Wallis H test or Mann–Whitney nonparametric *U* test. Differences in data between two families were examined using a *χ*^2^-test or Student’s *t*-test. Spearman correlation analysis was performed to determine differences in CD4^+^CD25^+^ Treg frequency and other parameters (serum ALT, TBiL, and CREA levels), with a *P* < 0.05 considered statistically significant.

## Results

### ACLF patients with increased frequency of CD4^+^CD25^+^ Tregs

**Figure 1:**
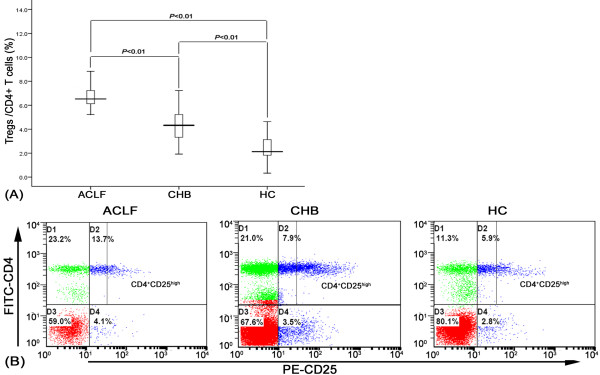
**Percentages of CD4**^**+**^**CD25**^**+**^**Tregs within total CD4**^**+**^**T population in peripheral blood in ACLF, CHB patients, and healthy controls.** The CD4^+^CD25^+^ Tregs are gated from a mononuclear cell subset of peripheral whole blood. (**A**) Frequencies of CD4^+^CD25^high^ Tregs in peripheral blood from three groups (ACLF, CHB patients, and healthy controls, HC). Data are expressed as box plots, in which the middle of the black solid line is the median frequency of Tregs. Vertical lines represent the 10^th^ and 90^th^ percentiles. The *p* values of multiple comparisons were calculated using the Kruskal–Wallis H nonparametric test. (**B**) Representative scatter plots are obtained by flow cytometry using antibodies against CD4 and CD25. CD4^+^CD25^+^ Tregs were separated into CD4^+^CD25^high^, CD4^+^CD25^low^ subsets, defined by the fluorescence intensity of CD25 (> 50) obtained using isotypic control antibody.

The CD4^+^CD25^high^ subset has been counted to represent the CD4^+^CD25^+^ Treg frequency in HBV and HIV-infected patients [22,23]. In the study, we thus determined the frequency of CD4^+^CD25^+^ Treg in total CD4^+^ by measuring CD4^+^CD25^high^ (fluorescence intensity of CD25 >50) population. Our results indicate that patients with HBV related-ACLF had a significantly higher frequency of peripheral CD4^+^CD25^high^ Tregs (mean, 6.24%) than CHB patients (mean, 4.16%, *P* < 0.01) and healthy controls (mean, 2.09%, *P* < 0.01). Moreover, there was a significant difference in the circulating proportion of CD4^+^CD25^high^ Tregs between CHB patients and healthy controls (*P* < 0.01) (Figure 1A). A representative flow plot demonstrating the frequency CD4^+^CD25^+^ Tregs in peripheral blood from ACLF patients, CHB patients and healthy controls is shown in Figure 1B.

### Relationship between Treg frequency and HBV DNA load

**Figure 2:**
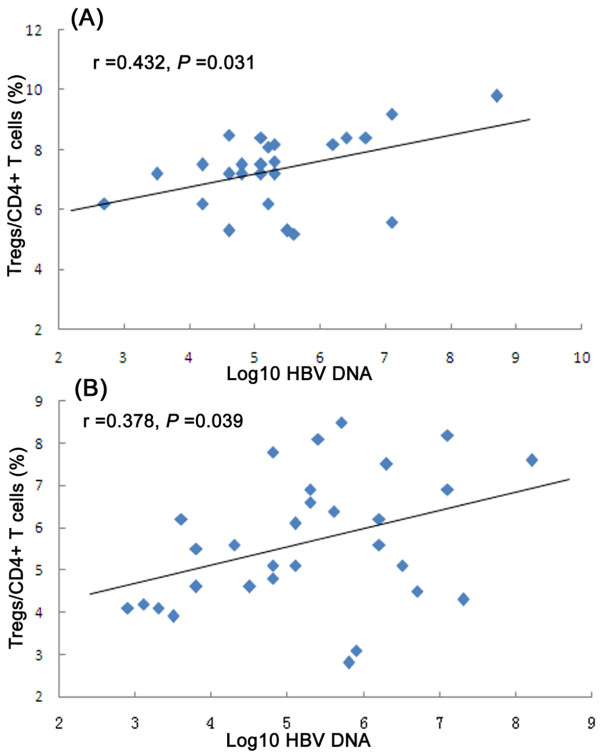
**Correlation between CD4**^**+**^**CD25**^**+**^**Treg frequency and common logarithm (log10) of HBV DNA load in patients with ACLF or CHB.**

There was a positive correlation between CD4^+^CD25^+^ Treg frequency in peripheral blood from ACLF patients and serum HBV DNA load (r = 0.432, *P* = 0.031) (Figure 2A). A similar positive correlation was also observed in CHB patients (r = 0.378, *P* = 0.039) (Figure 2B). Furthermore, in ACLF patients and CHB patients, there was no correlation between circulating CD4^+^CD25^+^ Tregs and serum ALT, TBiL, and CREA levels (data not shown).

### Tregs have a skewed TCRBV repertoire

**Figure 3:**
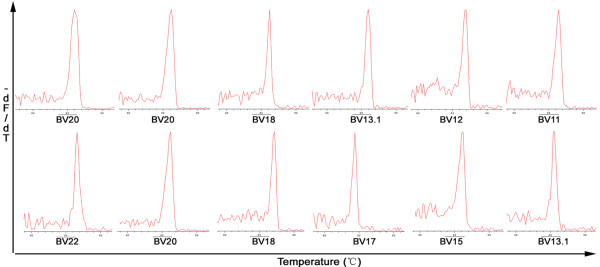
**Representative GMSP with a single-peak (monoclonal expansion) of TCRBV families in CD4**^**+**^**CD25**^**+**^**Treg from ACLF and CHB patients.** The TCRBV gene families in the top graphs corresponding to P43 (BV20), P70 (BV20), P51 (BV18), P46 (BV13.1), P25 (BV12), and P47 (BV11). The TCRBVs shown in the bottom graphs correspond to P20 (BV22), P17 (BV20), P57 (BV18), P45 (BV17), P17 (BV15), and P44 (BV13.1). The correspond amino acid sequences are shown in Table 3. The melting temperature is on the x-axis of each plot. The negative first derivative of the decrease in fluorescence as a function of temperature (−dF/dT) is shown on the y-axis.

We compared the Tregs TCRBV CDR3 profiles from ACLF and CHB patients to those of healthy controls. ACLF and CHB patients contained a greater number of skewed-clonally derived TCRBV families relative to controls. Although ACLF patients displayed a lower average ratio of skewed TCRBV families than CHB patients, it was not significantly different (*P* > 0.05) (Table 2). The number of single and biased-peaks for 24 TCRBV families detected in CD4^+^CD25^+^ Tregs differed between ACLF and CHB patients. Four TCRBV gene families (BV11, BV13.1, BV18, BV20) were more prevalent than other TCRBV members (Table 2). A representative GMSP profile of a monoclonal TCRBV expressed in CD4^+^CD25^+^ Tregs from ACLF and CHB patients is shown in Figure 3.

**Table 2:** **Frequency of skewed TCRBV families in CD4**^**+**^**CD25**^**+**^**Treg from patients with ACLF, CHB, and HCs**^**a**^

**TCRBV families**	**ACLF Incidence (%)**^**b**^	**CHB Incidence (%)**^**b**^	**HCs Incidence (%)**^**b**^
1	3	5	0
2	4	3	1
3	3	5	1
4	5	6	2
5.1	6 (26.1)	3	2
5.2	2	6	1
6	2	5	2
7	5	6	1
8	3	3	2
9	3	2	3
10	5	3	1
11	9 (39.1)	9 (33.3)	2
12	6 (26.1)	6	1
13.1	7 (30.1)	8 (29.6)	2
13.2	3	6	2
14	3	2	1
15	4	8 (29.6)	1
16	3	5	2
17	5	7 (25.9)	1
18	8 (34.8)	9 (33.3)	2
19	2	2	0
20	10 (43.5)	11 (40.7)	2
21	1	5	1
22	3	7 (25.9)	1
23	5	5	0
24	3	5	2
Total no. of skewed Vβ (average ratio for a case)	113 (4.91) ^c, d^	142 (5.26) ^c, d^	36 (2.12) ^c^
No. of patients examined with normal pattern ^e^ (ratio,%)	2 (8.0) ^f^	3 (10.0) ^f^	13 (43.3) ^f^
No. of patients examined	25	30	30

^a^The number of TCRBV gene families showing skewed-clone expansion (oligoclonal or monoclonal) is summarized in patients with ALCF, CHB and HCs.

^b^The number of samples showing a skewed-clone expansion in total detected samples (percentages of each TCRBV skewed-clone expansion). Samples with normal GMSP are excluded in the percentage calculation.

^c^The average skewed expansion rate of TCRBV gene families was higher in CD4^+^CD25^+^ Treg from patients with ACLF or CHB than that of HC group (*P* < 0.01 by *χ*^*2*^ test).

^d^There was no significant difference between the two groups (*P >* 0.05 by *χ*^*2*^ test). Samples with normal GMSP are excluded in the average ratio calculation

^e^Normal pattern (GMSP) means that there is no monoclonal or oligoclonal pattern in any TCRBV families in a patient.

^f^The incidence of the normal GMSP was significantly lower in CD4^+^CD25^+^ Treg from patients with ACLF or CHB than that of HC group (*P* < 0.01 by *χ*^*2*^ test).

### TCRBV CDR3 amino acid motifs in Tregs

The total number of TCRBV gene families with single peak shape (GMSP) in ACLF or CHB patients is 35 and 41 respectively. The TCRBV CDR3 gene families were cloned, sequenced, and translated to corresponding amino acid sequences. Representative amino acid sequences of TCRBV CDR3 from ACLF or CHB patients are shown in Table 3. We found that several different ACLF patients exhibited monoclonal expansion of relatively conserved TCRBV CDR3, such as BV20 (TGTGHSPLH with BJ1.6), BV18 (RTGDTEA with BJ1.1), BV13.1 (EVRTAFYEQ with BJ2.7) and BV11 (VYNEQ with BJ2.1). CHB patients also expressed relatively conserved TCRBV CDR3, such as BV20 (TGTGHSPLH with BJ1.6), BV18 (DDQETQ with BJ2.5), BV13.1 (YSGQGIDGY with BJ1.2) and BV11 (SSGGVDTQ with BJ2.3). Four TCRBV gene families (BV11, BV13.1, BV18, BV20) were more prevalent than the other TCRBV families.

**Table 3:** **Representative amino acid sequences of monoclonal TCRBV families in CD4**^**+**^**CD25**^**+**^**Tregs from patients with ACLF or CHB**

**Patients**^**a**^		**Vbeta**	**CDR3**	**BJ**		**Ratio**
43 (ACLF)	BV20	SGFYLCAWS	TGTGHSPLH	FGNGTRLTVTED	1.6	17/21
70 (ACLF)	BV20	SGFYLCAWS	TGTGHSPLH	FGNGTRLTVTED	1.6	19/23
67 (ACLF)	BV20	SGFYLCAWS	TGTGHSPLH	FGNGTRLTVTED	1.6	13/22
6 (ACLF)	BV20	SGFYLCAWR	ISHTGEL	LFGEGSRLTVLED	2.2	16/22
16 (ACLF)	BV20	SGFYLCAWS	GGSNQPQ	HFGDGTRLSILEDL	1.5	13/20
51 (ACLF)	BV18	SAAYFCASS	RTGDTEA	FFGQGTRLTVVED	1.1	16/21
46 (ACLF)	BV13.1	SVYFCASS	EVRTAFYEQ	YFGPGTRLTVTED	2.7	14/21
25 (ACLF)	BV12	SVYFCAIS	DGQVVEQ	YFGPGTRLTVTED	2.7	15/22
47 (ACLF)	BV11	SQYLCATG	VYNEQ	FFGPGTRLTVLED	2.1	20/20
51 (ACLF)	BV11	SQYLCATG	VYNEQ	FFGPGTRLTVLED	2.1	20/20
10 (ACLF)	BV5.1	SALYLCASS	LEQLTRANKQ	NFGPGTRLTVLED	2.3	17/23
20 (CHB)	BV22	SAMYFCASK	GSREGLVVNEQ	FFGPGTRLTVLE	2.1	18/22
17 (CHB)	BV20	SGFYLCAWS	TGTGHSPL	HFGNGTRLTVTED	1.6	21/23
57 (CHB)	BV18	SAAYFCASS	DDQETQ	YFGPGTRLLVLED	2.5	21/21
45 (CHB)	BV17	TAFYLCASS	IVNGGQPQ	HFGDGTRLSILED	1.5	21/22
17 (CHB)	BV15	TALYFCATS	DPASGRTNEQ	FFGPGTRLTVLED	2.1	15/22
44 (CHB)	BV13.1	SVYFCASS	YSGQGIDGY	TFGSGTRLTVVED	1.2	16/22
3 (CHB)	BV11	SQYLCATT	SSGGVDTQ	YFGPGTRLTVLED	2.3	21/23

When the GMSP profile of a TCRBV family displayed a single peak, the corresponding PCR products re-amplified and the CDR3 were sequenced after cloning. The amino acid sequences of the max rate of TCRBV families are shown. ^a^The clinical types of patients were indicated in brackets.

## Discussion

CD4^+^CD25^+^ Tregs are important immune regulatory cells. Their primary role is regulating the immune response through immune suppression by inhibiting the immune system response to both self and foreign antigens. Treg cells inhibit excessive pathological damage by inhibiting HBV-specific CD8 ^+^ T cell activation, which may aid persistent virus infection [8]. In addition to inhibiting CD4 ^+^/CD8 ^+^ T cells *in vivo*, Treg cells also inhibit dendritic cell (DCs) activation and cytokine secretion, preventing an excessive inflammatory response. We show here that increased prevalence of CD4^+^CD25^+^ Tregs in peripheral blood is associated with HBV replication and disease severity in HBV patients.

Several studies have tried to characterize the role of peripheral CD4^+^CD25^+^ Tregs in chronic HBV infection. However, the relationship between Tregs and ACLF pathogenesis is not well established, and in fact, the conclusions from two earlier studies were not consistent [11,12]. Our data are very similar to the findings observed by Xu et al. Those results support the notion that in HBV infected ACLF patients, up-regulation of CD4^+^CD25^+^ Tregs might play a critical role in suppressing immune responses, contributing to chronic HBV infection. In addition, the prevalence of circulating CD4^+^CD25^+^ Tregs is increased in CHB patients as described by Peng et al. [9]. Conversely, Wang et al. found a substantial decrease in peripheral CD4^+^CD25^+^CD127^low^ Tregs in ACLF patients. The discrepancies between these studies may be attributed largely to differences in techniques, reagents, and blood samples, as well as the molecular markers used for identifying Tregs.

HBV replication may play an important role in precipitating liver failure in patients with chronic HBV infection. Our results demonstrate a positive correlation between peripheral CD4^+^CD25^+^ Tregs and HBV DNA load in patients with HBV-related ACLF or CHB. Interestingly, adefovir treatment induced reduction of HBV DNA, a decrease in Tregs, and an increase in HBV-specific CD4^+^ and CD8^+^ T cell proliferation and IFN-gamma production [24]. These observations support the notion that an increase in CD4^+^CD25^+^ Treg may reduce HBV specific immune responses, leading to ongoing HBV replication and chronic infection.

T cells are quite heterogeneous due to the large repertoire of TCRs, which can serve as a “molecular fingerprint” of T cell populations [25]. Each T cell clone expresses a unique TCR that recognizes antigen-derived peptide bound to a major histocompatibility complex (MHC). The CDR3 region is the key determinant of T cell antigen specificity and mediates T cell diversity [26,27]. Therefore, analysis of the CDR3 profile reflects changes in the T cell population stimulated by a specific antigen [28,29]. In the current study, we found four TCRBV gene families (BV11, BV13.1, BV18, BV20) to be more prevalent than other members in CD4^+^CD25^+^ Tregs from ACLF and CHB patients, which suggests these four families may be indicative of patients with chronic HBV infection. Moreover, TCRBV5.1 is present at a higher frequency in ACLF patients than in CHB patients, which may indicate the TCRBV5.1 family may have a potential role in ACLF pathogenesis, although, this underlying mechanism requires further exploration.

Immune suppression plays an important role in the treatment of chronic hepatitis B. Inhibiting Treg proliferation and differentiation, blocking regulatory pathways, blocking immune suppression, and increasing epitope-specific CTL immune responses, are all potential new treatment strategies for hepatitis B [30,31]. At present, blocking T cell function using monoclonal antibodies against the TCR [32], and TCR gene transfer have been developed as a reliable method to generate large numbers of T cells ex vivo with given antigen-specificity and functional avidity. These results provide promise for clinical application [33-37].

In the current study, we found that motifs of monoclonal populations expressing TCRBV11, BV13.1, BV18, and BV20 in CD4^+^CD25^+^ Tregs from ACLF patients are different from that in CHB patients. It is not clear, however, if or how the emergence of the TCRBV families influence the course of ACLF. In a follow-up study, we observed an interesting phenomenon, wherein the TCRBV in CD4^+^CD25^+^ Tregs from ACLF patients expressed TCRBV20 with the CDR3 sequence “TGTGHSPLH”. Their short-term response to treatment was better than that for ACLF patients with the TCRBV CDR3 “ISHTGEL” or “GGSNQPQ” motifs. Moreover, we found the CDR3 “TGTGHSPLH” motif expressed only in the CHB patients whose condition was not as serious. This suggests that “TGTGHSPLH” may be a short-term prognostic biomarker for ACLF with anti-viral treatment, although this requires further validation. The TCRBV11 family prefers “VYNEQ” as a CDR3 motif in ACLF patients, but expressed “SSGGVDTQ” in CHB patients. This is partly consistent with our previous report showing “AGEL” is the preferred motif in CHB patients whose short-term outcomes are better than CHB patients expressing the “VYNEQ” motif [20].

Therefore, conserved TCRBV gene families may help produce antibody specific to TCRBV motifs, inhibiting the corresponding CD4^+^CD25^+^ Tregs and aiding hepatitis B treatment. Moreover, production of antigen-specific T cells using TCR gene transfer is a promising idea for the treatment of liver disease [38], with reports noting that CD8(+)CD45RC(low) Tregs are a potential cell-based treatment [39]. Examining CD4^+^CD25^+^ Treg TCR diversity may further our understanding of peripheral tolerance mechanisms and the role peripheral Tregs, and how this applies to hepatitis B patients [40].

In the current study, because the volume of blood samples was limited, the FoxP3 mRNA expression in CD4^+^CD25^+^ Tregs was not confirmed. Another hurdle is that CD4^+^CD25^high^ Tregs were not isolated using a cell sorter, and directly used for GMSP assay. But Peng et al. described that CD4^+^CD25^+^ Tregs could represent CD4^+^CD25^high^ Tregs, when the CD4^+^ T cells including high FoxP3-positive T cells content determined by flow cytometry analysis [9].

## Conclusions

Patients with HBV-related ACLF exhibited an increase in circulating CD4^+^CD25^+^ Tregs, which was correlated with HBV-related liver failure. An increase in peripheral Treg has been associated with more severe liver disease in hepatitis B patients. The relatively conserved TCRBV20 CDR3 “TGTGHSPLH” and TCRBV11 CDR3 “VYNEQ” motif could be used in predicting the health status of patients with ACLF or CHB, and may help in the development of transduction TCR gene therapy for ACLF patients.

## Abbreviations

ACLF: Acute-on-chronic liver failure; ALT: Alanine amino transferase; AST: Aspartate amino transferase; CHB: Chronic hepatitis B; FoxP3: Anti-forkhead family transcription factor 3; HBV: Hepatitis B virus; HBsAg: Hepatitis B surface antigen; HBeAg: Hepatitis B envelope antigen; Real-time-PCR: Real-time fluorescent quantitative polymerase chain reaction; GMSP: Gene melting spectral pattern; TBiL: Total bilirubin.

## Competing interests

The authors declare that they have no competing interests.

## Authors’ contributions

YJZ contributed to the study design, data collection, most experiments, and the writing the initial draft and revising the manuscripts. YP and WL collected the preliminary data, and helped to perform some experiments. XZR and CYB participated in the study design and interpretation of the data. TLL assisted in experimental design and help to data collection. LLJ contributed to the study coordination, technical issues and revision of the manuscript. All authors read and approved the final manuscript.
